# *Notes from the Field:* First Reported Case of *Shewanella haliotis* in the Region of the Americas — New York, December 2018

**DOI:** 10.15585/mmwr.mm6850a5

**Published:** 2019-12-20

**Authors:** Dakai Liu, Roberto Hurtado Fiel, Lucy Shuo Cheng, Takuya Ogami, Lulan Wang, Vishnu Singh, George David Rodriguez, Daniel Hagler, Chun-Chen Chen, William Harry Rodgers

**Affiliations:** ^1^Department of Pathology and Clinical Laboratories, NewYork-Presbyterian Queens, Flushing, New York; ^2^NewYork-Presbyterian Queens, Department of Surgery, Flushing, New York; ^3^Department of Dermatology, University of Pittsburgh Medical Center, Pittsburgh, Pennsylvania; ^4^Molecular Biology Institute, University of California, Los Angeles, California; ^5^Division of Infectious Diseases and Antimicrobial Stewardship, NewYork-Presbyterian Queens, Flushing, New York; ^6^Department of Pathology, Weill Cornell Medical College, New York City, New York.

On December 18, 2018, a man aged 87 years was evaluated in a hospital emergency department in Flushing, New York, for right lower abdominal quadrant pain. Evaluation included a computed tomography scan, which showed acute appendicitis with multiple abscesses measuring ≤3 cm. The patient was admitted, a percutaneous drain was placed, and 5 mL of an opaque jelly-like substance was aspirated and sent for culture and testing for antimicrobial sensitivities.

Gram stain of the culture revealed gram-negative rods, and culture revealed monomicrobial 1–2-mm yellowish-brown mucoid colonies.^†^ Sequencing of the isolate’s 16S ribosomal RNA revealed >99.8% homology with *Shewanella haliotis* strain DW01 in the GenBank database. Antimicrobial susceptibility testing indicated that the isolate was susceptible to aminoglycosides, fluoroquinolones, certain penicillins, and broad-spectrum cephalosporins (Table). Biochemical tests were performed to characterize isolate (Supplementary Table, https://stacks.cdc.gov/view/cdc/83522). Phylogenetic analysis indicates that *S. haliotis* strain DW01 is the most recent ancestor of this clinical isolate. This is the first documented case of a *S. haliotis* appendix infection.

**TABLE:** Antimicrobial sensitivity* of an isolate of *Shewanella haliotis* from an intraabdominal abscess — New York, December 2018

Antimicrobial	Drug class	MIC (*μ*g/ml)	Range			Interpretation
			S	I	R	
Amikacin	Aminoglycoside	3	≤16	32	≥64	S
Ampicillin	Penicillin	≤4	≤8	16	≥32	S
Ampicillin-Sulbactam	Penicillin-beta-lactamase inhibitor	>16/8	≤8/4	16/8	≥32/16	I
Aztreonam	Monobactam	>16	≤4	8	≥16	R
Cefazolin	Cephalosporin	>16	≤2	4	≥8	R
Cefepime	Cephalosporin	0.094	≤2	4–8	≥16	S
Cefoxitin	Cephamycin	>16	≤8	16	≥32	I
Ceftazidime	Cephalosporin	≤2	≤4	8	≥16	S
Ceftriaxone	Cephalosporin	≤1	≤1	2	≥4	S
Gentamicin	Aminoglycoside	0.25	≤4	8	≥16	S
Imipenem	Carbapenem	0.5	≤1	2	≥4	S
Levofloxacin	Fluoroquinolone	0.19	≤0.5	1	≥2	S
Meropenem	Carbapenem	0.047	≤1	2	≥4	S
Nitrofurantoin	Nitrofuran	>64	≤32	64	≥128	I
Piperacillin-Tazobactam	Penicillin-beta-lactamase inhibitor	≤2/4	≤16/4	32/4–64/4	≥128/4	S
Polymyxin B	Polymyxins	0.5	≤2	4	8	S
Tetracycline	Tetracycline	≤2	≤4	8	≥16	S
Tigecycline	Glycylcycline	0.38	≤2	4	≥8	S
Tobramycin	Aminoglycoside	0.5	≤4	8	≥16	S
Trimethoprim-Sulfamethoxazole	Dihydrofolate reductase inhibitor	0.5/9.5	≤2/38		≥4/76	S

**Abbreviations:** I = intermediate; MIC = minimum inhibitory concentration; R = resistant; S = sensitive.

* Quantitative determination of MIC conducted on Vitek 2 and Phoenix 100 testing systems. Sensitivity was interpreted according to Clinical and Laboratory Standards Institute/Food and Drug Administration guidelines.

*S. haliotis* is an emerging human pathogen, first isolated from abalone gut microflora in 2007 (*1*). The geographic distribution of human infections caused by *S. haliotis* is concentrated in Asia, with most reports coming from China, Japan, South Korea, and Thailand (*2*). No cases of *S. haliotis* human infections had been reported in the World Health Organization’s Region of the Americas.

The patient was treated empirically with intravenous piperacillin-tazobactam while in the hospital and was discharged with a prescription for oral amoxicillin-clavulanic acid. At a follow-up visit 13 days later, he was recovering well. Empiric treatment of *Shewanella* spp*.* can be challenging; limited and varying antibiotic susceptibility profiles have been reported (*2*,*3*). This patient’s isolate was susceptible to several classes of antimicrobials, but resistance to certain antibiotics has been observed in this isolate and others (*2*). In a case series of 16 patients from Martinique, *Shewanella* spp. sensitivities to piperacillin-tazobactam and amoxicillin-clavulanic acid were reported to be 98% and 75%, respectively (*3*).

Risk factors for or potential vectors of *Shewanella* spp. infections are unidentified in up to 40%–50% of cases (*4*). *S. haliotis* is ecologically distributed in marine environments, including broad contamination of cultivated shellfish. Although infection following consumption of seafood is seldom reported (*5*), consumption of raw seafood could be an important vehicle for foodborne illnesses and outbreaks. This patient reported consuming raw salmon 10 days before becoming ill but had no other marine exposures or exposure to ill contacts. The time from potential exposure to onset of abdominal pain in this patient is consistent with that reported in the literature on *Shewanella* spp. (3–49 days). The epidemiologic exposure history supports the link between raw fish consumption and infection.

No other organisms were isolated in this patient; in the Martinique case series of *Shewanella* spp*.*, one half of infections were monomicrobial as well (*3*). This case highlights the importance of preventing seafood-associated infections and the need to consider rare human pathogens in elderly or immunocompromised, marine-exposed populations, as well as persons who might consume at-risk food that might have been imported from outside the United States and persons who might have been infected outside the United States when traveling.

## Footnotes

* These authors contributed equally to the report.

† After inoculation of specimen on sheep blood agar, chocolate agar, and MacKonkey agar plates and incubation at 98.6°F (37°C), beta-hemolysis was observed on the blood agar plates.
